# Association of m.5178C>A variant with serum lipid levels: a systematic review and meta-analysis

**DOI:** 10.1042/BSR20212246

**Published:** 2021-12-17

**Authors:** Fuqiang Liu, Jiyun He, Shengping Wang, Feng Yu, Zhi Luo

**Affiliations:** 1 Department of Cardiology, First People’s Hospital of Chengdu, Chengdu, China; 2 Department of Nursing, Sichuan Nursing Vocational College, Chengdu, China; 3 Department of Internal Medicine, Zhongnan Hospital of Wuhan University, Wuhan, China

**Keywords:** coronary artery disease, epigenetics, lipid metabolism, m.5178C>A

## Abstract

**Background:** Emerging evidence shows that m.5178C>A variant is associated with a lower risk of coronary artery disease (CAD). However, the specific mechanisms remain elusive. Since dyslipidemia is one of the most critical risk factors for CAD and accounts for at least 50% of the population-attributable risk, it is tempting to speculate that the reduced CAD risk caused by the m.5178C>A variant may stem from an improved lipid profile. In order to verify this hypothesis, we conducted the present study to clarify the association of m.5178C>A variant with lipid levels.

**Methods:** By searching ten databases for studies published before 30 June 2021. Thirteen East Asian populations (7587 individuals) were included for the analysis.

**Results:** The present study showed that m.5178C>A variant was associated with higher high-density lipoprotein cholesterol (HDL-C) [standardized mean difference (SMD) = 0.12, 95% confidence interval (CI) = 0.06–0.17, *P*<0.001] and total cholesterol (TC) (SMD = 0.08, 95% CI = 0.02–0.14, *P*=0.01) levels. In subgroup analysis, the association of m.5178C>A variant with higher HDL-C levels were observed in Japanese (SMD = 0.09, 95% CI = 0.01–0.17, *P*=0.03) and Chinese populations (SMD = 0.13, 95% CI = 0.07–0.20, *P*<0.001). However, the association of m.5178C>A variant with lower low-density lipoprotein cholesterol (LDL-C) levels were only observed in Japanese populations (SMD = −0.11, 95% CI = −0.22 to 0.00, *P*=0.04).

**Conclusions:** The m.5178C>A variant was associated with higher HDL-C and lower LDL-C levels in Japanese populations, which may contribute to decreased CAD risk and longevity of Japanese.

## Introduction

The human mitochondrial genome is closely associated with the human lifespan, and each human cell contains hundreds to thousands of copies of the mitochondrial genome, which encode 13 polypeptides [1] that are associated with oxidative phosphorylation (OXPHOS) respiratory chain complex [2].

The m.5178C>A variant is rare worldwide [3] and is the earliest reported to be associated with longevity in Japanese populations [4]. Since variant of m.5178C>A primarily linked to Asian haplotypes [5] and relatively absent from European descents [6], this study was conducted only in East Asian populations due to a higher prevalence [4,5,7]. The m.5178C>A variant is located in the NADH dehydrogenase subunit-2 (*ND2*) gene of mitochondrial DNA (mtDNA) and formed by a transversion from cytosine (C) to adenine (A). The mtDNA-ND2 m.5178C>A variant leads to replacing leucine with methionine (p.Leu^237^Met).

A series of mouse model experiments [8–10] showed that the mtDNA damage affected lipid metabolism. For instance, the damaged mtDNA promoted lipid deposition in the atherosclerotic plaque [8], while the mtDNA dysfunction induced dyslipidemia [9]. Moreover, the loss of mitochondrial polymerase-γ proofreading activity (PolgD257A/D247A) and ApoE knockout (ApoE^−/−^) caused severe dyslipidemia [10]. Together, it indicated that the damage of mtDNA was associated with dyslipidemia. Notably, this speculation was verified by Sun et al. [11]. Moreover, an emerging study [12] reported that m.5178C>A variant was associated with improved mitochondrial functions. Intriguingly, the improvement of mitochondrial functions was associated with ameliorated lipid metabolism [13–15]. When combined with the above speculation, a variant of m.5178C>A may benefit lipid profile by enhancing mitochondrial functions.

Moreover, it was well documented that [4,7,16] variant of m.5178C>A was more frequent in centenarians than in general populations, suggesting that m.5178C>A variant was associated with longevity. Notably, this speculation was consistent with a complete mitochondrial sequencing analysis [17].

Over the last decade, intensive efforts [18–20] have been made in the scientific community to clarify the correlation between lipid metabolism and lifespan. The research results showed that the healthy [21] or the ameliorated [22,23] lipid profile may contribute to the extent of lifespan [21–23]. When combined with the above speculations whereby variant of m.5178C>A was associated with ameliorated lipid metabolism and longevity, it indicated that the ameliorated lipid profile might mediate the effects of the m.5178 variant on longevity.

The studies regarding the relationship between m.5178C>A variant and acute myocardial infarction (AMI) were limited but consistent. For instance, Mukae et al. [24] reported that the m.5178A>C variant was a risk factor of AMI. In contrast, Takagi et al. [25] claimed that the m.5178C>A variant was a protective factor of AMI. Moreover, Mitrofanov et al. [26] revealed that m.5178C>A variant was associated with reduced cardiovascular events in AMI patients. Although the m.5178C>A variant may reduce the AMI risk, the underlying mechanisms remain elusive. Therefore, the present study was required to clarify the effects of m.5178C>A variant on lipid levels and provide some clues or references to clarify possible mechanisms underlying m.5178C>A variant and AMI.

## Materials and methods

### Literature search

A comprehensive literature search was performed from 31 March 2021 to 30 June 2021 by using ten databases including PubMed, Medline, Embase, Cochrane Library, Web of Science, Google Scholar, Foreign Medical Journal Service, Excerpta Medica, CNKI, Wanfang. The following keywords were used in the search: (‘mtDNA’, ‘m.5178C>A’, ‘ND2-237’, ‘ND2-237 L/M’), (‘mutant’, ‘mutation’, ‘variant’, ‘variation’, ‘polymorphism’, ‘SNP’ or ‘single nucleotide polymorphism’) and (‘lipids’, ‘serum lipids’, ‘plasma lipids’, ‘circulating lipids’, ‘blood lipids’, ‘triglycerides’, ‘total cholesterol’, ‘low-density lipoprotein cholesterol’, ‘high-density lipoprotein cholesterol’, ‘TG’, ‘TC’, ‘LDL-C’ or ‘HDL-C’). Additionally, the reference lists of all eligible studies were manually retrieved to obtain more literature.

### Inclusion and exclusion criteria

The specific inclusion criteria were listed: (1) The test subjects must be limited to the East Asian population. (2) The studies investigated the association of m.5178C>A variant with serum lipid levels. (3) The studies at least provided one of four parameters in lipid profile (triglyceride (TG), TC, low-density lipoprotein cholesterol (LDL-C) and high-density lipoprotein cholesterol (HDL-C)). (4) The studies provided the frequency of m.5178C>A variant. (5) The studies offered the mean lipid levels with standard deviation (SD) or standard errors (SE) by m.5178C>A variant. (6) The interventional articles provided pre-intervention data. (7) The language of eligible studies was restricted to English or Chinese. The specific exclusion criteria were as follows: (1) The studies did not relate to m.5178C>A variant. (2) The studies did not relate to lipid levels. (3) The studies did not present the frequency of m.5178C>A variant. (4) The studies provided invalid data. (5) The studies provided incomplete data. (6) Pedigree studies. (7) Overlapping studies. (8) Abstract, review, case report, meta-analysis and animal studies.

### Data extraction

Two authors (Fuqiang Liu and Shengping Wang) extracted the data independently using a standardized data extraction table. The discrepancy in data extracted was resolved by consensus or a discussion with the third author (Zhi Luo). If critical data were absent, e-mail or telephone was used to contact the corresponding author to acquire this information. The following data were extracted from each eligible study: the last name of the first author, year, country (i.e., China and Japan), gender (males and females), ethnicity (i.e., Chinese and Japanese), the frequency of m.5178C>A variant, genotyping methods, type of study, type of disease, total sample size, mean lipid levels with SD or SE by m.5178C>A variant.

### Data analysis

The units of TG, TC, LDL-C and HDL-C were converted into mmol/l. All extracted data were expressed as mean ± SD. All the analyses were performed by STATA software (version 15.0, College Station, TX). *P*<0.05 was recognized as statistically significant. The standardized mean difference (SMD) and 95% confidence interval (CI) were used to evaluate the differences in lipid levels.

### Heterogeneity definition and processing

Inevitably, there were differences between the studies included in the meta-analysis. The differences or diversity between participants, interventions, and the *measurement results* among a series of studies were defined as ‘heterogeneity’ [27]. Heterogeneity was tested by *I^2^* statistic and Cochran’s χ^2^-based Q statistic. Galbraith plots were used to detect the potential sources of heterogeneity. If heterogeneity was significant (*I^2^* > 50%, *P*≤0.05), the random-effects model (DerSimonian–Laird method) was used to calculate the results [28]. Otherwise, the fixed-effects model (Mantel–Haenszel method) would be adopted. All synthetic results were recalculated after eliminating the studies with heterogeneity.

### Publication bias test

The publication bias among the included studies was evaluated by Begg’s funnel plot and Egger’s linear regression test [29].

### Subgroup analysis

Subgroup analysis was carried out by ethnicity, gender and health status. The ethnicity was divided into Chinese and Japanese. The health status was divided into type 2 diabetes mellitus (T2DM) patients and healthy subjects. In some studies, the subjects were divided into more than one subpopulation (e.g., the subjects originated from different races, the subjects with different types of disease, case and control subjects). Each subpopulation was regarded as an independent comparison in the present study.

### Sensitivity analysis

Sensitivity analysis was conducted in the present meta-analysis, in which the comparison was excluded one by one and performed the analysis again after omitting each comparison. If the synthetic results in any study changed substantially to alter the results from significant to non-significant or the other way around. The absence of such a phenomenon usually indicates the robustness and stability of synthetic results.

## Results

### Study selection

**Figure 1:**
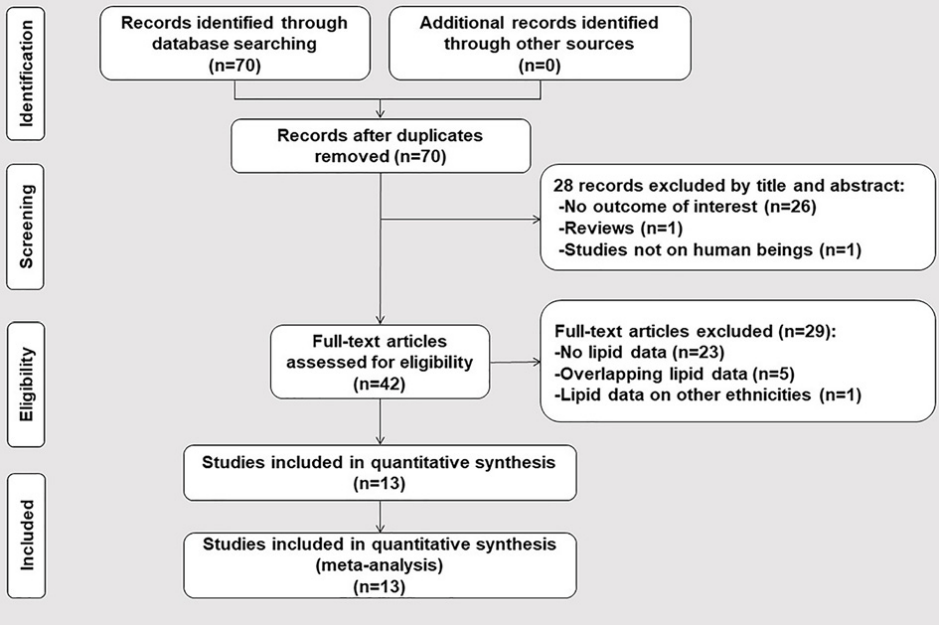
Flow diagram of the articles selection process A total of 70 studies were identified, and 28 studies were excluded by its title and abstract. A total of 42 possible studies were full-text reviewed and 29 studies were excluded because of absent lipid data (*n*=23), overlapping data (*n*=5), data from other ethnicities (*n*=1). Finally, 13 studies were included for quantitative analysis.

By searching the above ten databases, 70 studies were identified. After the screening, 28 studies were excluded by their title and abstract. Next, 42 studies were estimated by their contents. In which, 23 studies did not provide lipid data, 5 studies [30–34] had lipid data overlapping with other publications [35], and 1 study had lipid data of other ethnicities [36]. Therefore, 29 studies were further excluded. Finally, 13 studies (7587 individuals) were included in our study (Figure 1).

The references of the included studies were listed in Supplementary Material. The characteristics of the included studies were presented in Supplementary Table S1. The serum lipid levels by m.5178C>A variant were presented in Supplementary Table S2.

### Association of m.5178C>A variant with serum lipid levels

The outcomes of the analysis on all comparisons showed that m.5178C>A variant was associated with higher HDL-C levels (SMD = 0.12, 95% CI = 0.05–0.19, *P*=0.001) (Table 1).

**Table 1:** Associations of m.5178C>A variant with serum lipid levels

Groups or subgroups	Comparisons (Subjects)	*P*_H_	SMD (95% CI)	*P*_SMD_	Groups or subgroups	Comparisons (Subjects)	*P*_H_	SMD (95% CI)	*P*_SMD_
*Overall results*					*Recalculated results that eliminated heterogeneity*				
**TG**					**TG**				
All	17 (7477)	<0.001	−0.04 (−0.14–0.05)	0.36	All	13 (5918)	0.40	−0.05 (−0.11–0.01)	0.12
Japanese	10 (2388)	<0.01	−0.08 (−0.23–0.07)	0.30	Japanese	7 (1868)	0.44	−0.03 (−0.12–0.06)	0.54
Chinese	7 (5089)	0.02	−0.01 (−0.13–0.11)	0.85	Chinese	6 (4050)	0.24	−0.06 (−0.13–0.02)	0.12
Male	7 (1667)	0.35	−0.02 (−0.13–0.10)	0.78	Male	7 (1667)	0.35	−0.02 (−0.12–0.09)	0.78
T2DM	6 (3396)	0.01	0.01 (−0.21–0.23)	0.95	T2DM	5 (2357)	0.08	−0.10 (−0.26–0.06)	0.22
Healthy subjects	8 (1993)	0.02	−0.05 (−0.21–0.11)	0.53	Healthy subjects	6 (1600)	0.54	-0.03 (−0.14–0.07)	0.54
**TC**					**TC**				
All	16 (6789)	<0.001	−0.02 (−0.13–0.10)	0.79	All	12 (5782)	0.68	0.08 (0.02–0.14)	0.01
Japanese	9 (1700)	<0.001	−0.08 (−0.26–0.10)	0.37	Japanese	6 (867)	0.94	−0.05 (−0.19–0.08)	0.46
Chinese	7 (5089)	<0.01	0.05 (−0.08–0.19)	0.46	Chinese	6 (4915)	0.78	0.12 (0.05–0.18)	<0.01
Male	6 (979)	0.62	−0.01 (−0.14–0.13)	0.94	Male	6 (979)	0.62	−0.01 (−0.14–0.13)	0.94
T2DM	6 (3396)	<0.01	−0.04 (−0.33–0.25)	0.69	T2DM	4 (2810)	0.64	0.11 (0.02–0.19)	0.02
Healthy subjects	7 (1305)	0.15	0.06 (−0.10–0.21)	0.48	Healthy subjects	6 (1011)	0.61	−0.02 (−0.16–0.11)	0.72
**LDL-C**					**LDL-C**				
All	12 (6458)	0.42	0.01 (−0.04–0.07)	0.64	All	12 (6458)	0.42	0.01 (−0.04–0.07)	0.64
Japanese	5 (1369)	0.94	−0.11 (−0.22–0.00)	0.04	Japanese	5 (1369)	0.94	−0.11 (−0.22–0.00)	0.04
Chinese	7 (5089)	0.72	0.06 (−0.01–0.12)	0.08	Chinese	7 (5089)	0.72	0.06 (−0.01–0.12)	0.08
Male	5 (1481)	0.60	−0.09 (−0.20–0.02)	0.10	Male	5 (1481)	0.60	−0.09 (−0.20–0.02)	0.10
T2DM	5 (2984)	0.55	0.07 (−0.02–0.15)	0.11	T2DM	5 (2984)	0.55	0.07 (−0.02–0.15)	0.11
Healthy subjects	6 (1580)	0.74	−0.09 (−0.20–0.01)	0.09	Healthy subjects	6 (1580)	0.74	−0.09 (−0.20–0.01)	0.09
**HDL-C**					**HDL-C**				
All	18 (7587)	0.07	0.12 (0.05–0.19)	0.001	All	17 (7413)	0.24	0.12 (0.06–0.17)	<0.001
Japanese	11 (2498)	0.42	0.09 (0.01–0.17)	0.03	Japanese	11 (2498)	0.42	0.09 (0.01–0.17)	0.03
Chinese	7 (5089)	0.02	0.16 (0.04–0.27)	0.01	Chinese	6 (4915)	0.12	0.13 (0.07–0.20)	<0.001
Male	8 (1777)	0.54	0.13 (0.03–0.23)	0.01	Male	8 (1777)	0.54	0.13 (0.03–0.23)	0.01
T2DM	6 (3396)	0.01	0.15 (0.00–0.30)	0.05	T2DM	5 (3222)	0.05	0.11 (0.03–0.19)	0.01
Healthy subjects	9 (2103)	0.60	0.12 (0.03–0.21)	0.01	Healthy subjects	9 (2103)	0.60	0.12 (0.03–0.21)	0.01

Abbreviation: *P*_H_,* P*_Heterogeneity_.

Then the subgroup analysis was carried out (Table 1). Subgroup analysis by the characteristics of the subjects showed that the significant association of m.5178C>A variant with higher HDL-C levels was observed in Japanese, Chinese, T2DM patients, male and healthy subjects. However, the significant association of m.5178C>A variant with lower LDL-C levels was only observed in Japanese.

**Figure 2:**
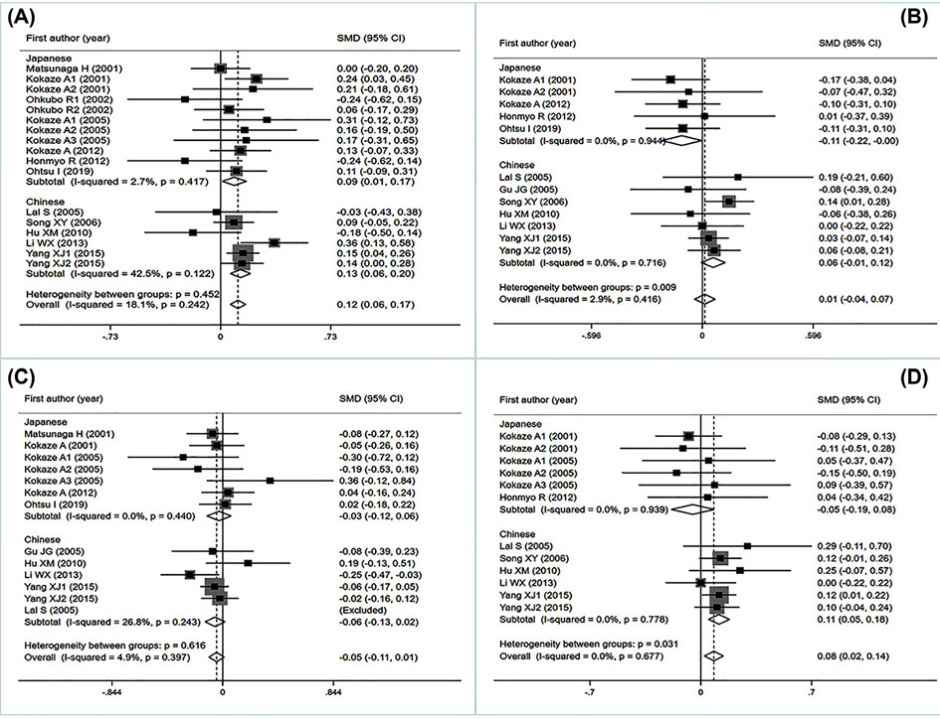
Forest plot of the meta-analysis between the mt5178 C/A mutant and serum lipid levels (**A**) mt5178 C/A and HDL-C levels. (**B**) mt5178 C/A and LDL-C levels. (**C**) mt5178 C/A and TG levels. (**D**) mt5178 C/A and TC levels.

The analysis that excluded the studies with heterogeneity was also carried out (Table 1). However, the significant associations of m.5178C>A variant with serum lipid levels did not change (Figure 2A–C) substantially excepting TC (Table 1 and Figure 2D), which showed statistical significance in Chinese and T2DM patients after eliminating heterogeneity.

### Evaluation of heterogeneity

In analyzing the association of m.5178C>A variant with lipid levels, significant heterogeneity was detected in TG, TC and HDL-C (Table 1). Four (Kokaze A2 2001, Ohkubo R1 2002, Ohkubo R2 2002, Song XY 2006), four (Matsunaga H 2001, Ohkubo R1 2002, Ohkubo R2 2002, Gu JG 2005) and one (Gu JG 2005) comparisons were recognized as the main contributors to TG, TC and HDL-C heterogeneity, respectively. The SMD values and 95% CIs of TG and HDL-C did not change substantially after excluding these comparisons (Table 1). However, the SMD value and 95% CI of TC (SMD = 0.08, 95% CI = 0.02–0.14, *P*=0.01) changed significantly after excluding these comparisons.

### Sensitivity analysis

**Figure 3:**
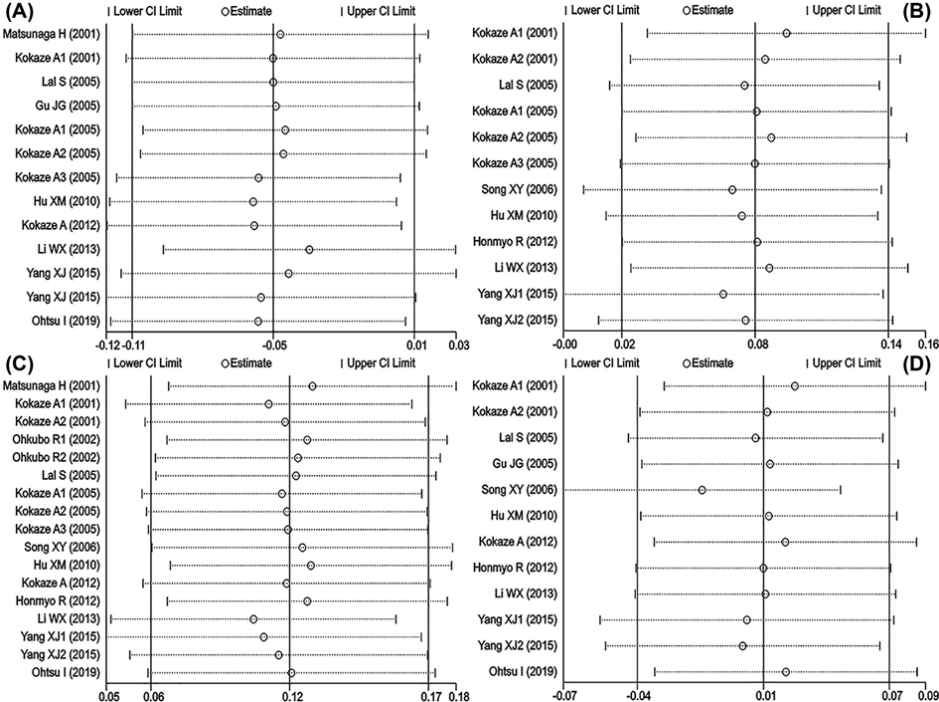
Sensitivity analysis between the mt5178 C/A mutant and serum lipid levels Open circle is SMID, parallel lines represent 95% CI. (**A**) mt5178 C/A and TG levels. (**B**) mt5178 C/A and TC levels. (**C**) mt5178 C/A and HDL-C levels. (**D**) mt5178 C/A and LDL-C levels.

Sensitivity analysis showed that no comparison might affect the association of m.5178C>A variant with TG (Figure 3A), TC (Figure 3B) and HDL-C (Figure 3C) levels. However, one comparison (Song XY 2006) may affect the significant association of m.5178C>A variant with LDL-C (Figure 3D) levels. Interestingly, the SMD value and 95% CI of LDL-C (SMD = −0.01, 95% CI = −0.08–0.05, *P*=0.66) did not change significantly after excluding this comparison.

### Publication bias test

**Figure 4:**
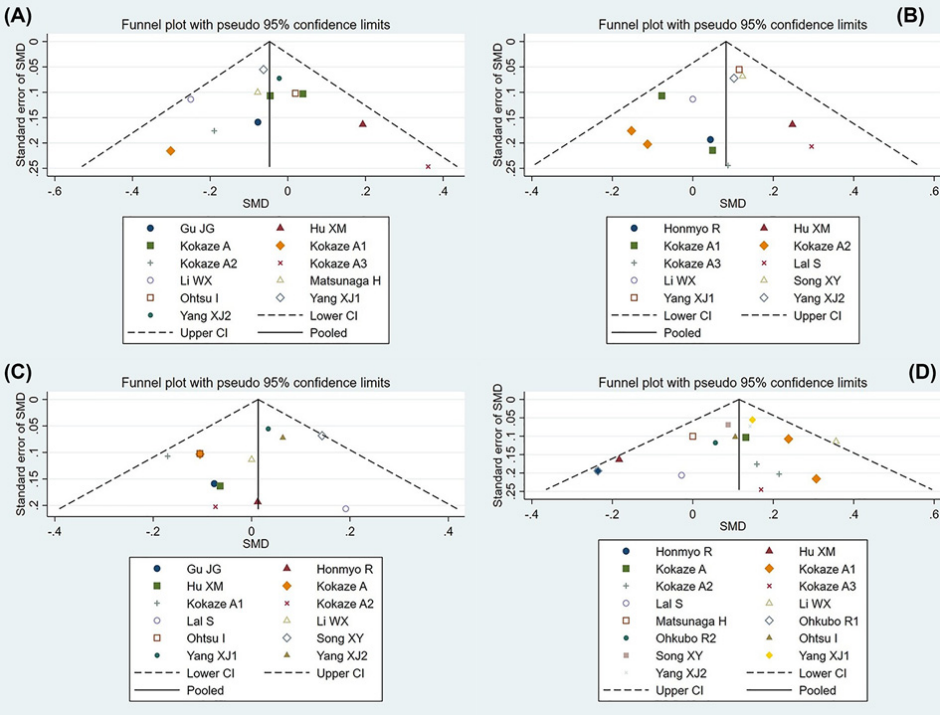
Begg’s funnel plot of the association analysis between the mt5178 C/A mutant and serum lipid levels The diverging lines represent 95% CI and the central line is SMD. (**A**) mt5178 C/A and TG levels. (**B**) mt5178 C/A and TC levels. (**C**) mt5178 C/A and LDL-C levels. (**D**) mt5178 C/A and HDL-C levels.

Begg’s funnel plot was used to evaluate the publication bias among the included studies. However, no publication bias was observed in synthetic results (Figure 4A–D), which was confirmed by Egger’s regression test (*P*=0.78 for TG, *P*=0.37 for TC, *P*=0.22 for LDL-C and *P*= 0.31 for HDL-C).

## Discussion

The present study showed that the m.5178C>A variant was significantly associated with higher HDL-C and TC levels. Subgroup analysis showed the significant association of m.5178C>A variant with higher HDL-C levels was primarily in Japanese and Chinese populations. While the significant association of m.5178C>A variant with lower LDL-C levels was only observed in Japanese populations.

The mechanisms underlying the association of m.5178C>A variant with lipid levels remain elusive. However, one hypothesis could be proposed to explain our findings, that is, by protecting against oxidative stress. The p.Leu^237^Met in mtDNA-ND2 may affect NADH dehydrogenase’s function and thus inhibit ROS release [37,38]. Moreover, a promising study conducted by Levine et al. [39] showed that the methionine readily reacts with oxidants to form methionine sulfoxide and thus scavenges oxidizing molecules [40]. Therefore, the increased methionine caused by m.5178C>A variant may protect against oxidative stress. Notably, this hypothesis was verified by an emerging study conducted by Tian et al. [12], in which, variant of m.5178C>A decreased ROS production and Caspase 3/7 activity, increased ATP production and membrane potential, and resistance to apoptosis. All these protective effects caused by this polymorphism demonstrated that variant of m.5178C>A was related to increased mitochondrial functions, which might benefit lipid metabolism [13–15,41–44].

In the present study, the significant association of m.5178C>A variant with higher HDL-C and TC levels was observed in Chinese (Table 1), indicating a contradictory correlation between m.5178C>A variant and lipid levels in Chinese populations. In addition, the significant association of m.5178C>A variant with higher HDL-C and lower LDL-C levels was observed in Japanese (Table 1), indicating a beneficial correlation between m.5178C>A variant and lipid levels in Japanese populations, which may contribute to the decreased susceptibility of AMI [24–26].

The mechanisms underlying the effects of m.5178C>A variant on longevity have not been fully clarified. However, it is now increasingly evidenced that the higher levels of HDL-C [22,45–47] and the lower levels of LDL-C [22,23] were associated with longevity. When combined with the present study, whereby m.5178C>A variant was associated with higher HDL-C levels and lower LDL-C levels in Japanese populations, this variant was associated with higher HDL-C levels in Chinese populations. It indicated that the higher HDL-C and lower LDL-C levels associated with m.5178C>A variant may contribute to the longevity of Japanese populations [4]. In contrast, the higher HDL-C levels associated with m.5178C>A variant may contribute to the longevity of Chinese populations [7].

Several limitations of the present meta-analysis should be noted. First of all, a large number of genes as well as some environmental factors are involved in dyslipidemia. However, this meta-analysis has not investigated the interactions of m.5178C>A variant with other variant loci or environmental factors on serum lipid levels due to the lack of original data from the included studies. Secondly, a relatively small number of individuals have been included in the lipid association analysis for m.5178C>A variant due to the limited number of studies that met the inclusion criteria, which may reduce the statistic power and even cause type I error (false-positive results). Thirdly, this meta-analysis only included the studies published in English and Chinese as it is very difficult to get the full papers published in various languages.

## Conclusions

The m.5178C>A variant was associated with higher HDL-C and lower LDL-C levels in Japanese populations, which may contribute to decreased coronary artery disease (CAD) risk and longevity of Japanese.

## Supplementary Material

Supplementary Tables S1-S2

Supplementary Material

## Data Availability

All data generated or analyzed during the present study are included in this article and its supplementary material.

## Abbreviations

AMI: acute myocardial infarction
ApoE^-/-^: ApoE knockout
CAD: coronary artery disease
CI: confidence interval
HDL-C: high-density lipoprotein cholesterol
LDL-C: low-density lipoprotein cholesterol
mtDNA: mitochondrial DNA
ND2: NADH dehydrogenase subunit-2
SD: standard deviation
SMD: standardized mean difference
TC: total cholesterol
TG: triglyceride
T2DM: type 2 diabetes mellitus
