# Supplementary material for: Association of m.5178C>A variant with serum lipid levels: a systematic review and meta-analysis
Source: Biosci Rep. 2021 Dec 17;41(12):BSR20212246. doi: 10.1042/BSR20212246 (PMC8685646; doi:10.1042/BSR20212246)
Supplement: Supplementary Tables S1-S2 [file BSR-2021-2246_supp.pdf]

**Supplemental Tables**

**Table S1.** Characteristics of the studies included in the meta-analysis.

| **First author [reference]** | **Year** | **Ethnicity** | **Gender** | **Study population** | **Total sample size** | **Outcomes** |
|---|---|---|---|---|---|---|
| Matsunaga H [1] | 2001 | Japanese | M/F | Patients with type 2 diabetes mellitus | 412 | TG, TC, HDL-C |
| Kokaze A1 [2] | 2001 | Japanese | M | Healthy subjects | 362 | TG, TC, LDL-C, HDL-C |
| Kokaze A2 [2] | 2001 | Japanese | F | Healthy subjects | 99 | TG, TC, LDL-C, HDL-C |
| Ohkubo R1 [3] | 2002 | Japanese | M/F | Patients with cerebral hemorrhage or infarction | 127 | TG, TC, HDL-C |
| Ohkubo R2 [3] | 2002 | Japanese | M/F | Healthy subjects | 294 | TG, TC, HDL-C |
| Lal S [4] | 2005 | Chinese | M | Healthy subjects | 211 | TG, TC, LDL-C, HDL-C |
| Gu JG [5] | 2005 | Chinese | M/F | Patients with type 2 diabetes mellitus | 174 | TG, TC, LDL-C, HDL-C |
| Kokaze A1 [6] | 2005 | Japanese | M | Healthy subjects | 91 | TG, TC, HDL-C |
| Kokaze A2 [6] | 2005 | Japanese | M | Healthy subjects | 138 | TG, TC, HDL-C |
| Kokaze A3 [6] | 2005 | Japanese | M | Patients with obesity | 67 | TG, TC, HDL-C |
| Song XY [7] | 2006 | Chinese | M/F | Patients with type 2 diabetes mellitus | 1039 | TG, TC, LDL-C, HDL-C |
| Hu XM [8] | 2010 | Chinese | M/F | Patients with type 2 diabetes mellitus | 220 | TG, TC, LDL-C, HDL-C |
| Kokaze A [9] | 2012 | Japanese | M | Healthy subjects | 394 | TG, TC, LDL-C, HDL-C |
| Honmyo R [10] | 2012 | Japanese | M | Healthy subjects | 110 | TG, LDL-C, HDL-C |
| Li WX [11] | 2013 | Chinese | M/F | Patients with type 2 diabetes mellitus | 448 | TG, TC, LDL-C, HDL-C |
| Yang XJ1 [12] | 2015 | Chinese | M/F | Patients with type 2 diabetes mellitus and control subjects | 1894 | TG, TC, LDL-C, HDL-C |
| Yang XJ2 [12] | 2015 | Chinese | M/F | Patients with type 2 diabetes mellitus | 1103 | TG, TC, LDL-C, HDL-C |
| Ohtsu I [13] | 2019 | Japanese | M | Healthy subjects | 404 | TG, TC, LDL-C, HDL-C |

M: male; F: female; TG: triglycerides; TC: total cholesterol; LDL-C: low-density lipoprotein cholesterol; HDL-C: high-density lipoprotein cholesterol.

**Table S2.** Serum lipid levels by the m.5178C>A variant.

| First author, reference | Number | | TG, mmol/L | | TC, mmol/L | | LDL-C, mmol/L | | HDL-C, mmol/L | |
|---|---|---|---|---|---|---|---|---|---|---|
| | **C** | **A** | **C** | **A** | **C** | **A** | **C** | **A** | **C** | **A** |
| Matsunaga H [1] | 243 | 169 | 1.78±0.88 | 1.69±1.47 | 5.02±0.6 | 4.86±0.64 | - | - | 1.42±0.24 | 1.42±0.27 |
| Kokaze A1 [2] | 214 | 148 | 1.46±0.7 | 1.43±0.71 | 5.29±0.88 | 5.22±0.89 | 3.21±0.83 | 3.06±0.85 | 1.41±0.37 | 1.50±0.38 |
| Kokaze A2 [2] | 55 | 44 | 1.23±0.52 | 0.96±0.52 | 5.72±0.83 | 5.63±0.84 | 3.51±0.77 | 3.45±0.78 | 1.65±0.41 | 1.74±0.41 |
| Ohkubo R1 [3] | 89 | 38 | 1.4±0.52 | 1.11±0.3 | 5.44±0.95 | 4.8±1.1 | - | - | 1.23±0.31 | 1.15±0.4 |
| Ohkubo R2 [3] | 164 | 130 | 1.16±0.37 | 1.27±0.44 | 5.44±0.71 | 5.66±0.74 | - | - | 1.52±0.37 | 1.54±0.34 |
| Lal S [4] | 184 | 27 | 1.64±0 | 1.68±0 | 5.41±1.25 | 5.79±1.53 | 3.51±1.31 | 3.76±1.27 | 1.28±0.36 | 1.27±0.35 |
| Gu JG [5] | 113 | 61 | 1.54±0.83 | 1.48±0.67 | 4.68±0.73 | 4.21±0.9 | 2.52±0.49 | 2.48±0.59 | 0.89±0.26 | 1.08±0.48 |
| Kokaze A1 [6] | 55 | 36 | 1.34±0.63 | 1.15±0.63 | 5.25±0.81 | 5.29±0.82 | - | - | 1.54±0.46 | 1.68±0.45 |
| Kokaze A2 [6] | 86 | 52 | 1.44±0.69 | 1.31±0.68 | 5.3±0.98 | 5.15±0.99 | - | - | 1.41±0.31 | 1.46±0.32 |
| Kokaze A3 [6] | 34 | 33 | 1.59±0.72 | 1.85±0.72 | 5.26±0.8 | 5.33±0.8 | - | - | 1.25±0.29 | 1.3±0.3 |
| Song XY [7] | 737 | 302 | 1.92±1.18 | 2.13±1.63 | 5.42±1.24 | 5.57±1.14 | 1.3±0.33 | 1.35±0.39 | 3.09±0.91 | 3.17±0.92 |
| Hu XM [8] | 172 | 48 | 1.56±0.87 | 1.72±0.66 | 4.58±1.15 | 4.89±1.57 | 2.43±0.8 | 2.38±0.71 | 1.23±0.46 | 1.15±0.34 |
| Kokaze A [9] | 239 | 155 | 1.54±1.03 | 1.58±1.03 | - | - | 3.14±0.9 | 3.05±0.79 | 1.41±0.35 | 1.46±0.42 |
| Honmyo R [10] | 64 | 46 | - | - | 5.24±0.94 | 5.28±0.87 | 3.17±0.83 | 3.18±0.77 | 1.41±0.3 | 1.34±0.29 |
| Li WX [11] | 348 | 100 | 2.1±1.2 | 1.8±1.2 | 4.3±0.9 | 4.3±1 | 2.4±0.8 | 2.4±0.7 | 1.2±0.3 | 1.3±0.2 |
| Yang XJ1 [12] | 1472 | 422 | 1.57±1.32 | 1.49±1.12 | 4.65±1.14 | 4.78±1.08 | 2.66±0.87 | 2.69±0.96 | 1.24±0.4 | 1.3±0.42 |
| Yang XJ2 [12] | 857 | 246 | 1.75±1.36 | 1.72±1.26 | 4.49±1.18 | 4.61±1.14 | 2.71±0.94 | 2.77±0.96 | 1.14±0.42 | 1.2±0.43 |
| Ohtsu I [13] | 247 | 157 | 1.55±1.03 | 1.57±1.02 | - | - | 3.14±0.89 | 3.05±0.8 | 1.41±0.35 | 1.45±0.42 |

TG: triglycerides; TC: total cholesterol; LDL-C: low-density lipoprotein cholesterol; HDL-C: high-density lipoprotein cholesterol.
