# Supplementary material for: Association of m.5178C>A variant with serum lipid levels: a systematic review and meta-analysis
Source: Biosci Rep. 2021 Dec 17;41(12):BSR20212246. doi: 10.1042/BSR20212246 (PMC8685646; doi:10.1042/BSR20212246)
Supplement: Supplementary Material [file BSR-2021-2246_supp1.pdf]

**Supplemental References**


1. Matsunaga H, Tanaka Y, Tanaka M, Gong JS, Zhang J, Nomiyama T, Ogawa O, Ogihara T, Yamada Y, Yagi K, Kawamori R. Antiatherogenic mitochondrial genotype in patients with type 2 diabetes. Diabetes Care. 2001;24:500-503.
2. Kokaze A, Ishikawa M, Matsunaga N, Yoshida M, Sekine Y, Teruya K, Takeda N, Sumiya Y, Uchida Y, Takashima Y. Association of the mitochondrial DNA 5178 A/C polymorphism with serum lipid levels in the Japanese population. Hum Genet. 2001;109:521-525.
3. Ohkubo R, Nakagawa M, Ikeda K, Kodama T, Arimura K, Akiba S, Saito M, Ookatsu Y, Atsuchi Y, Yamano Y, Osame M. Cerebrovascular disorders and genetic polymorphisms: mitochondrial DNA5178C is predominant in cerebrovascular disorders. J Neurol Sci. 2002;198:31-35.
4. Lal S, Madhavan M, Heng CK. The association of mitochondrial DNA 5178 C > a polymorphism with plasma lipid levels among three ethnic groups. Ann Hum Genet. 2005;69:639-644.
5. Gu JG, Zhou X, Li X, Li D, Liu SH, Hu PP. Association of the Mitochondrial DNA5178A / C Polymorphism with Serum Lipid Levels in Patients with Type2Diabetes Mellitus. Journal of new medical knowledge. 2015; 15:40-44.
6. Kokaze A, Ishikawa M, Matsunaga N, Yoshida M, Makita R, Satoh M, Teruya K, Sekiguchi K, Masuda Y, Harada M, Uchida Y, Takashima Y. Longevity-associated mitochondrial DNA 5178 C/A polymorphism is associated with fasting plasma glucose levels and glucose tolerance in Japanese men. Mitochondrion. 2005;5:418-425.
7. Song XY, Lu B, Dong XH, Ding D, Hu RM. Association of the mitochondrial DNA 5178 A/C polymorphism with diabetic nephropathy in Chinese type 2 diabetic patients. Fudan UnivJ Med Sci. 2006; 33: 479-482.
8. Hu XM, Lv JX. Study on the association of the mitochondrial DNA5178C / A polymorphism with type2diabetes mellitus. Lab Med Clin. 2010; 7: 1820-1822.
9. Kokaze A, Ishikawa M, Matsunaga N, Karita K, Yoshida M, Ohtsu T, Ochiai H, Shirasawa T, Saga N, Hoshino H, Takashima Y. Combined effect of longevity-associated mitochondrial DNA 5178 c/a polymorphism and green tea consumption on risk of hypertension in middle-aged Japanese men. Hum Biol. 2012;84:307-318.
10. Honmyo R, Kokaze A, Karita K, Yoshida M, Ishikawa M, Ohno H. Influence of mitochondrial DNA 5178 C/A polymorphism on serum cholesterol changes: a short-term follow-up in middle-aged Japanese men. Environ Health Prev Med. 2012;17:401-407.
11. Li WX, Wu H, Lv JX. Assessment of mitochondrial DNA ND2 gene C5178A polymorphism for cerebral-cardiovascular complications in patients with type 2 diabetes mellitus. Clin J Lab Med. 2013; 36: 519-522.
12. Yang X, Zhang Y, Ma Y, Zhao Q, Lyu J. Effect of mitochondrial DNA 5178 C/A polymorphism on risks for type 2 diabetes mellitus and its complications. Zhonghua Yi Xue Yi Chuan Xue Za Zhi. 2015;32:855-860.
13. Ohtsu I, Ishikawa M, Matsunaga N, Karita K, Yoshida M, Ochiai H, Shirasawa T, Yoshimoto T, Minoura A, Sai S, Kokaze A. Longevity-associated mitochondrial DNA 5178 C/A polymorphism modifies effect of aging on renal function in male Japanese health checkup examinees: an exploratory cross-sectional study. J Physiol Anthropol. 2019;38:12.
